# Intersect between self-esteem and emotion regulation in narcissistic personality disorder - implications for alliance building and treatment

**DOI:** 10.1186/s40479-017-0054-8

**Published:** 2017-02-07

**Authors:** Elsa Ronningstam

**Affiliations:** 1 Harvard Medical School, Belmont, USA; 2 McLean Hospital, AOPC Mailstop 109,115 Mill Street, Belmont, MA 02478 USA

**Keywords:** Narcissistic personality disorder, Emotion regulation, Self-esteem regulation, Trauma, Attachment, Alliance

## Abstract

Building an alliance with patients with pathological narcissism or narcissistic personality disorder, NPD, can be challenging and include avoidance, negative reactivity and disruptions. A main contributing factor can be the complex interaction between emotion and self-esteem regulation, which affects patients’ ability to engage in a therapeutic alliance and treatment. Recent studies, especially in neuroscience have identified functional characteristic and compromises in self-esteem and emotion regulation related to NPD. Self-enhancement, hyper reactivity and need for control, which patients within the range of disordered narcissism often present, can have different roots and underpinnings that require thorough exploration in the process of building the therapeutic alliance and promote change in treatment. Clinical examples with treatment implications and strategies will be discussed to highlight both internal fluctuations and external features and shifts in narcissistic personality functioning.

## Background

Patients with pathological narcissism or narcissistic personality disorder, NPD, are difficult to engage in treatment and tend to drop out early. Building an alliance with these patients can be a tenuous and challenging endeavor. Nevertheless, a collaborative alliance is necessary for treatment to be working and changes to occur, independently of treatment modality [1–3]. Disagreements and disruptions are common and can readily lead to stalemates or premature termination. Sometimes disagreements can be quite apparent; directly verbally and emotionally expressed, more or less confrontational, but yet within relatively obvious and direct interactions between therapist and patient. Other times expressions of disagreements can be less noticeable; resulting in mismatch and disconnection, or even remain hidden, unexpressed and seemingly absent.

Therapists’ confusion, and vivid countertransference or personal reactions can in these situations be informative at best, but often distracting or misguiding [4, 5]. The discrepancy between the patients’ verbal communication and their internal mindset with convictions and vulnerabilities can be difficult to discern. We therapists tend to make hypothesis or conclusions either based on the patients’ obvious striking appearance and verbosity, or on assumptions about the patients’ internal thoughts and intentions. Those may be theoretically well anchored but may still not match the patients’ actual experiences in the moment, or concur with what they are ready and able to access and process in the alliance with the therapist. The therapists find themselves “talking at” the patient, and unable to reach and connect with the patient. Similarly, therapists often feel that the patient is “talking at” them. One contributing factor to these challenges with narcissistic patients can be the complex interactions between their emotion and self-esteem regulation that affect their ability to engage in a mutual collaborative alliance.

The main aim of this paper is to discuss how emotion regulation and self-esteem intersect, co-vary and mutually affect each other in narcissistic personality functioning. This interdependence is also explored in the context of patients’ different levels of and fluctuations in their functioning. Of interest is both how patients’ functioning can affect emotion and self-esteem regulation, and the reverse, how functioning can be affected by emotion and self-esteem regulation, separately and by their co-interaction. Of specific interest is how the interpersonal interactive expressions and patterns of fluctuating self-esteem and affects regulations can unfold in the alliance with the therapist, especially in the phase of alliance building, but also during ongoing treatment. Rather than labeling and categorizing phenotypic functioning, clinicians and therapists are encouraged to help the patients access and explore the specific individual nature of their internal triggers and fluctuations, and how they are expressed in interpersonal interactions. Of additional interest is how these fluctuations also can relate to the influence of life circumstances as well as of treatment interventions.

Fluctuations with sudden shifts can occur at any time during the treatment. Consequently, enhancement, spurred by aggression or fear and accompanied by detachment or dismissiveness, can readily shift to inferiority and insecurity, accompanied by avoidance or a sense of loss of control caused by overwhelming shame, fear or powerlessness. These fluctuations can be especially informative as the alliance building approaches critical and challenging aspects of the patients narcissistic functioning. In addition, they can also inform about earlier established attachment patterns and crucial developmental events or trauma that are reactivated and unfolding. This can occur at any point in treatment and independently of treatment modality.

This conceptualization of narcissistic pathology is anchored in a dimensional approach to narcissism and NPD as well as in studies that support the co-occurrence and fluctuation of different phenotypical expressions of self-enhancement and fragility within each individual patient [6]. It aims at directing clinicians’ and therapists’ focus beyond their own negative reactions and countertransference, as well as away from the patients’ immediate and often strikingly engaging or provocative attitudes and reactions. Those situations easily invite initial counteractive interventions which further tend to activate patients’ narcissistic pathology and objections to treatment. Maneuvering, negative collisions and detachment occur at a stage when patients usually are not ready, i.e., not in a sufficient agential juncture to be able to mobilize motivation, connectivity, curiosity and reflective ability to engage in the gradually evolving and deepening interactive therapeutic process. This paper will discuss an approach that serves to balance the patient’s resistance and defensive reactivity while pursuing therapeutic explorations and interventions that attend to narcissistic pathology.

First, recent relevant studies, especially in neuroscience, will be reviewed that identify and differentiate several functional characteristic and compromises in narcissistic patients’ emotion regulation, including difficulties identifying, tolerating, verbalizing and processing feelings, and the effect of secondary emotions on sense of control, motivation and self-esteem. Second, different interactional patterns will be discussed that are influenced both by compromised emotion processing and by fluctuations in self-esteem regulation in these patients. The overt self-enhancement, hyper reactivity, and need for control that patients within the range of disordered narcissism often present can have different roots and underpinnings, which require thorough exploration in the process of building the therapeutic alliance and promote change in treatment. Third, clinical examples with treatment implications and strategies will be discussed that serve to identify coexisting and fluctuating self-enhancement and vulnerability that are affected by the interplay between both self-esteem and emotion regulation, as well as by attachment patterns and external life events and circumstances.

### Regulation of self-esteem in NPD

Self-esteem and sense of self-worth can be affected by several factor and circumstances in patients with pathological narcissism or NPD [7–9]. External markers in social, professional, physical, financial or material contexts can be perceived as evidence of self-worth, while loss or lack thereof can cause a more or less drastic sense of defeat or worthlessness. Shifts in experiences of interpersonal affiliations and attention, from being included, appreciated and admired to being excluded, criticized and ignored; can be extremely challenging for the individual’s self-esteem. Similarly, sense of internal control, with consistent ability to predict and understand contexts, emotions, interactions and intentions, are essential for self-esteem and sense of agency [10]. This is often translated into evidence of competence with more or less consistent accomplishments and success. Sense of self-esteem can also be interpersonally assessed, primarily in emotional or relational performance or social maneuvering. In that context, narcissistic patients’ agential capacity can be an essential catalyst, not only for interpersonal competence but also for self-enhancing manipulation and interpersonal control.

In addition, social dominance and leadership [11] can for some people with pathological narcissism be a very important base for self-esteem, a motivational source that can provide attention, and sense of competence and control. Consequently, experiencing loss of control and competence can evoke intense internal self-criticism, with accompanying shame, anxiety, rage or fear, and result in drastic actions to regain or to escape the situation. Finally, and as mentioned above, emotions, both others or own, can be either awarding, challenging or threatening, mainly depending upon how they are perceived by the patients and how they affect their self-esteem, and sense of competence and control.

Self-esteem in patients with NPD can be sturdily enhanced and fragilely inferior, with influence of insecurity. These differences have foremost been phenotypically identified as separate prototypes of NPD, i.e., “thick-skinned” and “thin-skinned” [12, 13], “overt” and “covert” [14], and “grandiose” and “vulnerable” [6, 15]. In addition, Kernberg [16] introduced a dimensional view on narcissistic personality functioning. Conceptualized in terms identity, defenses and reality testing on three levels of personality organization: neurotic, borderline and psychotic, a range of severity in narcissistic personality functioning could be identified. Accordingly, patients with NPD can range from functioning on a lower neurotic or higher level of borderline personality organization (BPO) with some degree of integration of self and others, flexible defenses and capacity for reality testing and ability to work, i.e., with more sturdy potentials for self-esteem regulation. On the middle and low levels of BPO functioning, such patients show identity diffusion, lack of integration of self, variable reality testing, primitive defenses, and inability to maintain work and love relations, sometimes combined with antisocial personality disorder, psychopathy or malignant narcissism. The foundation for these patients’ self-esteem regulation is much more compromised and involving a high degree of brittle and/or severely pathological strategies.

However, as mentioned above, recent studies have observed the co-occurrence and internal interactions between sturdiness and stability vs fragility and fluctuation. In particular, the balance and fluctuations between enhanced and inferior self-esteem can vary significantly both within each individual as well as between the different prototypes of NPD [17]. Some can have a more robust consistent sense of their self-esteem at work, but can also recognize that in other situations, such as those more social or unstructured, they can feel vigilant, vulnerable and insecure. Others can be highly achieving, ambitious and successful with smooth and well controlled social skills, and at the same time be relatively unaware of their areas of fragility, like in more intimate relationships. Such patients can become extremely reactive to and frightened by threats or sudden loss of self-esteem. Still others can be preoccupied with a constant effort to maintain or achieve desirable levels of self-esteem using different more or less conscious external maneuvers and strategies while experiencing a rollercoaster like internal struggle with negative emotions and critical undermining self-judgments. Identifying the contextual influence on individual self-esteem regulation has also been important [18]. The impact of life events and changes, especially those sudden unexpected or consequential, can severely rupture a narcissistic individual’s self and self-esteem regulatory pattern including both enhancing and avoiding/protecting strategies, leading to loss of functioning and unfolding of more or less severe narcissistic pathology and even suicidality [19].

Studies of explicit and implicit self-worth and self-esteem (conscious vs autonomic/unconscious evaluation of the self) in NPD patients tend to support these complex interactions with foremost consistently vulnerable versus acutely threatened or damaged self-esteem. Vater and colleagues [20], in one of the very few studies that included patients clinically diagnosed with NPD who also were inpatients, confirmed the vulnerable low explicit self-esteem in NPD, and that its co-occurrence or “collision” with high implicit self-esteem causes high symptom severity in NPD. Of specific interest is how comorbid conditions and occurrence of hospitalization can affect NPD patients’ self-esteem, and vice versa, how their self-esteem fluctuation and accompanying functioning can make hospitalization necessary. High explicit self-esteem can accompany specific capability and functioning in some patients with NPD for periods of time, even if they have comorbid conditions (e.g., substance dependency or eating disorder). On the other hand, states of depression or recurrent anxiety can in and by itself cause low explicit self-esteem. It is therefore important to discern whether the circumstance or condition leading up to hospitalization, or the hospitalization by itself is the actual cause of a reduction in the patient’s explicit self-esteem. For some NPD patients a hospitalization can be a corrective emotional event leading to a realization that they indeed need treatment. For others, however, it may be the reverse, and instead escalate such shame and negative reactions that prevent patients’ motivation and involvement in further treatment.

### Regulation of emotions in NPD

Similar to self-esteem, the experiences and regulations of emotions can be affected by different factors in patients with NPD [21]. Alexithymia refers to the inability to feel and identify own feelings, either due to unawareness or to incapacity to distinguish physical and affective states, or because of lack of words for emotions [22, 23]. This inability or deficit contributes to significant emotion dysregulation in people with NPD, as well as to difficulties identifying, understanding and processing others’ feelings. Hypervigilance with sensitivity, reactivity and negative affects especially in response to humiliation or other challenging or traumatizing events is associated with pathological narcissism [24]. However, avoidance can also be motivating, i.e., a defensive self-regulatory strategy to avoid failure, which helps to protect the fragile self-esteem [25].

The ability for care and empathy can fluctuate and depend upon both emotion regulation and self-esteem [26]. Compromised, but not a lack of empathic capability can result in a range of interpersonal responses to others’ needs and reactions; from total ignorance, avoidance or dismissive or even aggressive responses, to extraordinary attentiveness and care in contexts where such engagement also is associated to self-enhancement and possible benefits.

People with NPD can appear unaffected by losses, separation or experiences that normally would evoke sadness, pain and anguish. They have even been considered unable to grieve [27]. Nevertheless, their extreme hyper vigilance and reactivity to certain threats, separations or losses of people or conditions that are crucial for their self-esteem, are also notable. Drastic impulsivity, violence or deadly suicidal acts can follow [19, 28].

Aggression can serve several different functions in pathological narcissism and NPD [22, 29–33]. It can be protective and enhancing; incorporated in ambitions, perfectionism, and proactive competitiveness and dominance, as well as in exceptional competency as an underlying motivating energy. It can also be dismissive or callous as obvious in interpersonal condescending, critical obnoxious and rejecting behavior that negatively influence intimate, social and professional relationships. The degree of controlled versus reactive more or less impulsive aggression can vary among narcissistic individuals. Aggressive reactions in response to real or perceived threats, humiliation or insults can be more common in patients with fragile or fluctuating self-esteem, and self-enhancement with readily triggered insecurity. On the other hand, those with stable and consistent self-enhancement with compartmentalized insecurity or vulnerability are more “thick-skinned” [13] and less prone to such reactivity. When aggression is directed towards the self, it is expressed in self-criticism, devaluation or self-hatred. Such aggression can also be an internal force in suicidal ideations or actions. However, when such ideations remain chronic and not turned into action, they can actually serve to maintain the patients’ sense of internal control and paradoxically help preserve their connection to life [34]. In more severely pathological or malignant narcissism, aggression can become seriously destructive or revengeful with controlled manipulativeness or impulsive actions [28, 29].

Shame is a prevalent and complex emotion in NPD that can be either explicit conscious as well as implicit subconscious or unconscious. Feelings of shame that relate to explicit external or other-directed attributions tend to evoke shame-based aggressive, critical or blaming reactions, while implicit shame, which is especially associated to the self, can drive more consistent self-enhancing regulatory strategies including perfectionism and competitiveness [35]. Shame is associated with anticipation or actual experiences of failure and negative exposure, especially in perfectionist and success oriented people. Shame can contribute to underlying fragility and hypersensitivity in narcissistic personality functioning. It can also contribute to difficulties in emotion processing, and motivate both avoidance as well as reactive defensive, retaliatory anger to regain agency and control [36–38].

Fear can underlie several management and avoidance strategies typical for NPD, including achievement and competitiveness, perfectionism, risk-taking, procrastination, and distancing and avoidance. The fear of negative experiences or intolerance of self or certain aspects of the identity can enforce protective self-enhancement as well as despair and potential suicidality [39]. Similarly, fear associated with early, especially narcissistic trauma can be reactivated in the presence. The balance between fear and motivation to process is especially challenging in treatment when approaching and working though trauma and fear reactivating conflicts. This requires a certain level of trust in the therapeutic alliance, as well as the patient’s courage and sense of agency [40, 41].

### Studies of emotion regulation and pathological narcissism

Recent research on recognition, processing and control of emotions in subjects with pathological narcissism or NPD provide further evidence and indications of compromised or fluctuating abilities in emotion regulation. Marissen and colleagues found less accuracy in recognizing emotional expressions in others, especially those related to feelings of fear and disgust [42] and Sagar and Stoeber [36] found difficulties in emotion processing caused by feelings of shame. In an fMRI study by Fan and colleagues [23] subjects were presented pictures of emotional faces and asked to empathize with the person in the picture. They noticed shifts in narcissistic subjects from inter- to intra-subjective relationship with increased focus on self when processing of emotional faces. This related to difficulties assessing both own and others’ emotions as well as to difficulties simulating others affects states.

Additional studies have suggested both neurological and psychophysiological cores for internal control and emotion regulation in patients with NPD. One study by Sylvers and colleagues [43] found sympathetic activation and negative reactions to happy stimuli, and indifference to fearful and sad stimuli, which suggest a psychophysiological base for narcissistic emotion regulation. Other studies identified deficits in structural brain functioning in NPD, i.e., the prefrontal grey matter (GM) volume that affect emotion regulation and emotional empathic processing, which suggest a neurological core for noticeable fluctuations in NPD patients’ self-regulation and control of emotions [44, 45].

### Etiological factors in NPD

Additional factors for consideration when approaching self-esteem and emotion regulation in alliance building relates to the multifactorial etiology of NPD, which form deeply ingrained patterns for patients’ self-regulation and interpersonal relating. These include inheritance [46], temperament [47], psychological trauma [40, 48], and age inappropriate role assignments [49], which all can contribute to a personality functioning that is characterized by hypersensitivity, shame, aggressivity, self-esteem enhancement and fluctuations, and identity diffusion. Early attachment patterns are especially formative: *dismissing* i.e., contemptuous derogation and/or brittle idealization of attachment figures; *preoccupied* attachment especially to objects who can enhance esteem and control; *anxious* and *avoidant*, related to narcissistic vulnerability; or *cannot classify* with multiple, unintegrated attachment alternating between dismissing, devaluating and angry or overwhelmed preoccupation [50–54]. Especially when multiple and/or unintegrated attachment patterns co-occur with fluctuations in emotions and self-esteem regulation, the patient may present with a very challenging and provocative interpersonal and reactive pattern in treatment.

Although more apparent for others these patterns may be less accessible or even inaccessible to the patient’s awareness and would need a carful and gradual exploration and flexibility in choices of treatment modality and interventions.

Experiences of trauma, especially those of psychological as opposite to physical nature, are common developmental causes to pathological narcissism and NPD, also perpetuated and affecting the adult personality functioning [40, 48]. These traumas are usually caused by intermittent neglect, sudden loss of idealized attention or relationships, severely humiliating experiences, or terrifying or fear-provoking incomprehensible experiences, beyond the patients’ age appropriate expectations and comprehensive capability, leaving them unsupported and alone to process and come to terms with what they encountered. Such traumas can be deeply hidden, under dismissive or avoidant attachment patterns and be more or less effectively defended against. In fact, narcissistic pathology can have a stabilizing effect that supports personality functioning and shields the effects of early trauma [51]. Often embedded in deep shame, these traumas may not be readily verbalized and accessible for communication. The reactivation and re-experience of these traumas, with accompanying intense and overwhelming emotional experiences and fluctuations in self-esteem, can be a motivating factor for patients to seek treatment. It can also, if occurring in ongoing treatment, require adjustments or considerations to add additional treatment modalities focused specifically on processing of trauma.

### The interaction between self-esteem and emotions

Shifts in self-esteem can evoke intense emotions that sometimes can be intolerable and difficult to process for patients struggling with pathological narcissism. A former executive told his therapist:

*“I was demoted when my company merged with another company. A colleague of mine got the position I had expected to get and prepared myself for. I decided to end my life because I could not see a future for myself in the new merged company. When I woke up after my failed suicide attempt I had to deal with all the shame, humiliation, worthless and envy I felt.”*

Similarly, intense emotions can affect self-esteem and cause sudden changes, either towards enhancement or towards decrease or loss with accompanying insecurity and inferiority. An example is a mother of 3 children who told her therapist:

*“I can’t stand my children’s emotional reactions and demands on me. It makes me feel so inadequate. It is just painful and overwhelming. I prefer when they play sports or perform. That reflects well on me, and can shine in the light of their accomplishments and be appreciated as a mother.”*

Self enhancement and control, often so striking in patients with NPD and an inevitable obstacle in treatment, can have different causes and underpinnings, and it can be challenging for therapists to find a collaborative opening that allows further explorations.

*Case vignette #1*

*T: “With your substance use history you are obviously at high risk for relapse”*

*Pt: “No I am not !! How dare you say that to me? Your comment really puts me at risk for relapse!!!! I don’t want to see you ever again!*

This patient, a woman in her late 20s and with a long history of substance use and a diagnosis of NPD, and with several efforts in the past to gain and maintain sobriety that inevitable ended in relapses, had now been sober for 4 months. This time she was facing an ultimatum from her grandfather; if she did not maintain sobriety and move on with her life and career, she would lose a substantial inheritance. She had a fragile sense of identity as sober, with complex self-esteem fluctuations, and intense reactions to perceived intrusions that could imply faults or suggest assigned intentions. This caused a sense of loss of control and being overpowered, which resulted in aggressive defensiveness with various self-enhancing strategies including drugs. By pointing out the risk for relapse the therapist obviously activated a more dismissive attachment and reactivity pattern that closed the door for further collaborative exploration and possibilities of reaching a shared understanding of the patient’s motivations and experiences related to both substance use and progression in her life.

An alternative strategy would be the following:

*T: “What do you think could make you want to resume substance use?*

*Pt: “I don’t intend to pick up substance use again!!!”*

*T: “I understand that, but it may be useful for us here to be aware of what made you resume drugs in the past so we together know what can affect you now in that direction.*

*Silence.*

*Pt: “I see you point…. well I know that loosing boyfriends and failing at tests have been very devastating for me. But drugs also made me feel very good and in control, especially of my feelings, and I clearly miss that, but I can also see that it would be a failure for me if I resume substance usage, and this time it also will have serious negative consequences for me. Before, it really did not matter.*

By choosing to start with an open question the therapist invited the patient to further collaborative exploration about her awareness of the context and reasons for relapses in the past. The patient’s initial rejecting response did not end the alliance but opened an opportunity for the therapist to further clarify her incentive. Obviously that engaged the patient’s own motivation to share her recollections of experiences, and she revealed both gaining and losing self-esteem with substance usage, and the role of substance as a vehicle for gaining inner control and regulation of intolerable emotions. Facing the present ultimatum from her grandfather obviously opened further realizations of purpose and consequences in her choices.

There are several advantages with using inquires, open questions and general statements that invite different perspective in the alliance building phase with NPD patients. First, the therapist can engage the patient’s sense of agency, which can encourage rather than challenge the patient’s sense of internal control and self-esteem. At the same time it can gradually activate the patients’ curiosity, reflective ability and narratives of their own experiences and perspectives. Second, through inquiries the therapist also gets important insight and understanding of the patient’s own experiences and reasoning. This enable the therapist to move beyond the patient’s striking enhancing or defensive behavior and reasoning, which may verify the diagnostic criteria for NPD but usually provide less of an informative foundation to build a therapeutic alliance with the patient and work towards change in personality functioning. Third, the exploration can unfold the patient’s complex interdependence between emotion regulation and self-esteem fluctuation. In this case vignette #1, both enhancement and vulnerability in self-esteem and accompanying emotions could unfold in the alliance with the therapist, and specifically in the context of substance abuse. The mutual awareness of these fluctuations between therapist and patient is crucial for the therapeutic process to move forward towards integration of the patient’s sense of self with less need for protective and dismissive maneuvers.

*Case Vignette #2*

*Pt: I hate my boss, he is the most stupid, ineffective idiot I know. I get so stressed out in his presence. He is focused on all these details…. calls for reports that have to be delivered within a couple of hours. Yesterday, I was on an important Skype meeting finalizing a large business deal, and he suddenly interfered with his ridiculous requests. I just can’t stand him and it is not good for my mental health. I am afraid that I will lose my temper and that would not be good for my career.*

*T: So you feel intimidated by your boss*.

*Pt: No I don’t, you obviously do not understand… My boss is an obstacle for my promotion and continuing career. He does not want to see me advance in the company; he does not appreciate my contributions and he only wants the numbers for his reports.*

*T: Hmmmm …..That can be a challenging situation in a corporate workplace….*

*Pt: Yes, I appreciate that you see that, I will be perceived as a failure by my colleague if I don’t move on, and this is really humiliating for me. I have worked hard in my career and work means a lot for me, I don’t have anything else in my life, my wife left me a few years ago and I am responsible for paying my children’s college tuitions although they don’t live with me and I hardly ever see them.*

In this case the patient presents with emotion dysregulation in an interpersonal context, more specifically in interaction with a superior in the workplace. The therapist’s initial intuitive choice of intervention focused on reformulating the patient’s experiences in terms of a narcissistic humiliation. That evoked the patient’s critical, defensive reactions and he quickly outlined a defensive self-enhanced victimization scenario that potentially also could be perceived as somewhat suspicious or even paranoid. The patient also demonstrated a preoccupied attachment style as played out in relationship to his boss. However, the therapist’s shift to validate the situation as “challenging”, without further intentions or emotional attributions, changed the interaction. The patient became more trusting and revealed a complex and difficult internal experience related to a challenging life context, which he described foremost in terms of struggles related to self-esteem; demands, expectations, anticipations of failure, etc., and of the perception of his boss as the immediate obstacle for his preservation of self esteem and ability to fulfill life responsibilities, as well as for his aspirations and advancements. This was also a pattern that had occurred earlier in his life and career.

There are several advantages of using general descriptive statements as therapeutic interventions, such as “challenging”, “difficult”, “tricky”, “complex” etc., especially in the phase of alliance building. Such more neutral and agency engaging statements that acknowledge the patients’ struggle, also tend to lessen their reactive and dismissive defensiveness, and instead activate their ability to provide more informative descriptions of their difficult experiences related to both self-esteem and emotions. In this case vignette #2 the patient’s initial emotional reactivity turned out to be rooted in a complex and lonely life situation with real demands, aspirations, and preoccupation with concerns about potential failures. The interaction between narcissistic self-enhancement and emotion regulation exemplifies escalating aggression in a primarily self-esteem threatening situation with underlying vulnerability, including fear, shame, loneliness, and negative anticipations of losing control and failing to live up to expectations, hopes and aspirations. It shows the close interchange between actual competence, enhancement and fragility, and between splits and projections. Attending to both the patient’s internal subjective experiences as well as his interpersonal enactments (both vis-à-vis boss and therapist) is important but require that the therapist can balance multiple layers of observations before making choices for the focus for interventions. With this patient an initial focus on his self-esteem and related internal subjective experiences enhanced his sense of agency and reflective ability. An alternative interpersonal approach with focus on the patient’s emotional reactivity, either in the initial disconnect in the alliance between patient and therapist, or in relationship between the patient and his boss, would readily be experienced as blame by the patient, since his core issue primarily related to self-esteem that caused the accompanying emotional reactivity. This also requires the therapist’s close attention to own countertransference reactions, real personal reactions, theoretical approach, and past experiences [55].

### Compromised emotion processing and regulation

Patients with NPD tend to present with confusing and sometimes quite disorganized emotional and empathic functioning. Based on the review of empirical findings regarding emotion regulation above, the therapist’s task to identify contexts and causes of a patient’s emotion processing can be quite challenging and interventions can readily be erroneous and cause breaches in the alliance. Narcissistic patients can have problems with different aspects and range of emotion regulation: ability to feel a feeling; tolerate the nature and/or intensity of a feeling; identify and verbalize a feeling; identify and translate physiological/visceral expressions of an affect, such as breading, heartbeat, dizziness, tension, cramps, pain, etc., into emotional experiences that can be verbalized and communicated to others; or with integrating own feeling and intentions into interpersonal interactions that are congruent with personal intensions, moral/ethical values and social/cultural conventions [21]. Motivation, willfulness and deliberate enhancing or defensive manipulative communication can also persuade narcissistic patients’ interaction. In addition some patients with NPD can present in a social and friendly manner with exceptional verbal plasticity, and with an ability to integrate appropriate intentional vocal tone and phrasing of emotions that may be more or less disconnected from their internal genuine emotional experiences.

*Case vignette #3*

*A father of a 17 year old son, described feeling very guilty for not attending to his son and helping him with his homework and papers. The therapist noticed a discrepancy between the patient’s verbal expression of profound guilt and an obvious comfort and assurance in his appearance and facial expression.*

*T: what do you think prevents you from helping your son?*

*Pt: really nothing ……I am just busy with work right now, I have a very important project.*

*T: That can be a tough dilemma for a father….*

*Pt: Yep…..I suppose so…..but that is just how it is now!*

*The therapist sensed an underlying difficulty in the patient’s rater matter of fact description of the conflicts in his role as a father that potentially could involve much deeper and more complicated experiences. However, the patient closed the subject and the therapist made a note of its importance with a hypothesis that the patient was not ready to discuss it further at that moment.*

*A couple of months later.*

*Pt: you asked me what prevented me from helping my son, and I referred to my work as I usually do, but it is much more complicated than I wanted to admit. I used to feel that I was a good father when my son was young. He looked up to me and needed me. I felt I could help him and we had fun together. But as he grew older I sensed he got increasingly critical of me; pointed out my mistakes and criticized me openly in front of friends and relatives. I have to admit, I don’t understand his homework, and he makes me feel inferior. I can’t stand it. I have learned to say that I feel guilty as a father because I spend so much time at work, and that has been helpful because people get sympathetic and supportive, but in reality I can't tolerate my son’s physical and intellectual development. He has become sooo much smarter, taller and stronger than me. The perspective of him surpassing me athletically and professionally should be a source of pride, but it is not, it makes me afraid and I just want to run away.*

*T: that is a challenging situation, maybe you had wished that I too, like your friends, just had been sympathetic and understanding of your guilt.*

*Pt: well it would have been easier, but I suppose I am here to attend to the real difficulties. And there is more to it that relates to my experiences with my own father as well as with difficulties with my mother.*

During the following months the patient gradually revealed a series of complicated experiences of his large, detached and intermittently critical, aggressive and frightening (psychologically abusive) father who left the family when he was 15. The patient was then left to take care of a younger sister and his mother, who struggled with cancer. The patient had developed well as an adult, committed to sobriety after a period of substance use, advanced in his professional career, got married and raised a son. Although he preferred solitude, he had learned to be appropriately social and interactive. His smooth verbal and interactive style had served him well both socially and professionally; an example of how narcissistic pattern of avoidance with self-enhancing strategies, social adaptability and professional skills can provide a functional base, at the same time as is can cover impaired and constrained emotional functioning and insecurity related to underlying trauma. The traumatic memories of the father and accompanying fear, shame and inferiority had remained encapsulated and unprocessed under his self-focused achievement oriented, competitive functioning. The son’s development had gradually unfolded the patient’s traumatic developmental experiences. They could unexpectedly escalate, causing moments of role reversal with shockingly frightening self-esteem fluctuations and threatening intense emotions.

This case vignette demonstrates a patient’s intertwined self-esteem and emotional dysregulation, with an initial predominantly avoidant attachment pattern and an inability to address the deeper traumatic roots that were affecting his present functioning. It also demonstrates the co-occurrence of both enhancement, actual competence and fragility within the same individual, and how fluctuations suddenly can occur, both in the patients’ life context and in the interaction within the treatment alliance. The regulatory range from actual ability to defensive or aggressive self-enhancement and to insecurity with inferiority, shame fear and loss of sense of agency is notable.

Early trauma that suddenly reoccurred and interfered with the patient’s pride and abilities escalated the patient’s narcissistic pathology. Trauma can promote a false ego organization with specific efforts to handle loss of ideals and parental protection. Paradoxically, certain aspects of NPD, such as fantasies of omnipotence, martyr ship, control and primitive prettified guilt, can actually give meaning to and help take charge of horrifying early narcissistically traumatic experiences [40]. When reoccurring, the trauma can cause an acute internal state that threatens the individual’s continuity, coherence, stability, and wellbeing. Narcissistic processes aimed at organizing and understanding the original traumatic experience fail. The sense of loss, rejection, and abandonment, along with feelings of shame, fear, and worthlessness, can, like with this patient, become overwhelming [56, 57].

The therapist’s respect for the patient’s initial rejection to engage in further explorations of the underpinnings to the manifest problem of guilt for not helping his son actually enabled an opening later in the alliance building. At that point the patient apparently felt more trust and/or agential courage, and could initiate the subject and take charge of a narrative that unfolded his deeper, complex, emotionally frightening and traumatic experiences. The therapist could also at that time point out the initial discrepancy in the alliance, i.e., the patient’s wish for the therapist to be supportive and helpful with the patient’s feelings of guilt for not being a good enough father, versus the therapist’s inviting inquiry about underlying contributing reasons for avoiding paternal care and responsibilities, and experiences that he failed as a father. This represented a processing of the initial transference development made possible in the deepening stage of the alliance. The patient’s readiness for a new realization of the necessity to address deeper and frightening issues, and the therapist pointing out and containing both the patient’s wish for his support as well as his call for further explorations and entering much more challenging experiences, could at that point be incorporated in the alliance. The patient’s gain of self-esteem and sense of agency in the alliance was not just an indication of a defensive maneuver but a necessary vehicle for being able to address his intolerable emotional experiences associate both to present life context as well as to past developmental experiences and severe emotional trauma. It is, however, important to balance and attend to the real defensive aspects of the patient’s narcissistic pathology that indeed can have helped him move on and establish a relatively well structured adult live. Taking on age inappropriate parental responsibilities early in life enforces certain self-enhancement and mastering of underlying fear and insecurity. Similarly, it enforces regulation of emotions, compartmentalization and avoidance.

## Treatment implications

When starting treatment with patients with pathological narcissism or NPD and engaging them in alliance building (independently of treatment modality) it is important to identify the roots and underpinnings of each patient’s narcissistic self-regulation in general, and of specific situational self-esteem and emotion fluctuations in particular. Is the patient primarily struggling with compromised abilities and deficiencies, such as intolerance of or compromised emotion processing, discrepancies between verbal and executive cognitive abilities, or neuropsychological compromised functioning, which can be expressed as avoidance or self-centeredness in response to emotionally or interpersonally challenging situations? Or is the patient struggling with residual effects of psychological trauma or early formed attachment patterns of dismissive, avoidant, or preoccupied nature? An additional possibility is that the patient is not ready and fully motivated for a genuine engagement, but feels more or less forced or mandated to attend treatment.

### Differentiate motivation and neurological ability

Identifying signs of and differentiating between patients lack of or fluctuating motivation, versus compromised or lack of neurologically based ability to relate and engage in processing within a therapeutic modality, is crucial [26]. The neurological studies as mentioned above suggest increased self-focus, negative reactions to emotional stimuli, difficulties to accurately recognize emotions, and compromised ability for internal control in NPD patients. In addition, co-morbid attention deficits can affect the narcissistic patient’s executive functioning. Especially when co-occurring with self-enhancing, dismissive or avoidant narcissistic traits and patterns, such deficits can cause extraordinary challenges in alliance building [58]. All this requires therapists’ careful identification and exploration of patients’ functioning and a thorough differential diagnosis before deciding and applying an intervention. For example, a patient refused to attend an assigned group therapy, stating that she could not tolerate the intense emotions expressed by the other group members and consequently she felt accused when both the group leader and the participants told her she “lacked empathy” and was trying to control the group. This escalated the patient’s aggression and urges to retaliate. Further exploration in the therapy, using a non-judgmental approach and some psycheducation, clarified that when the patient was exposed to other group members’ emotional reactions, sadness and frustration as well as joy or happiness, it evoked such overwhelming, intolerable arousal and tension, which made the patient feel incompetent, exposed, envious, and extremely vulnerable. Neuropsychological testing further confirmed this dysregulatory tendency. Such awareness and evidence about the patients functioning suggested the need for different treatment interventions and approaches that took into consideration the patient’s compromised emotion processing and intense reactivity, and engaged the patient’s motivation for self-regulatory strategies, self-reflective ability, and sense of agency.

### Competence, agency and self-esteem regulation

Based on the discussion above, it is also important to gradually differentiate patterns related to self-esteem fluctuations from those that primarily are triggered by emotion dysregulation. The patient’s emotions can be deeply entangled under a surface of self-enhancement and aggressive defensive reactivity, and this requires a gradual and balanced exploration to sort out their interactive patterns and implement changes. The patient’s hyper vigilance, manipulation and reactivity, especially in the initial phase of alliance building, can often relate to reluctance, shame or even fear of entering treatment. A focus on self-esteem rather than emotions, such as validation of the challenges that evolve in the treatment, can invite patients’ proactive agency and encouraging a more solid and trusting base for initiating a treatment alliance that gradually can contain and process more intense emotional interactions. Similarly, acknowledging the patient’s actual and reality anchored skills and competence (vocational/professional, psychological, physical, intellectual, social etc.,) is important, especially as they readily by the patient can be turned into or by the therapist be perceived as primarily self-enhancing or manipulating. In addition, patients can present with different self-regulatory directions: toward interpersonal withdrawal with self-preoccupation and dismissiveness or avoidance, or toward interpersonal preoccupation with more or less forceful competition, criticism, maneuvering, or even toward attentive compliance with readiness to support and agree with the therapist. An exploratory approach with non-judgmental curiosity and interest in the patient’s emotional reactions, i.e., noticing that the patient suddenly avoids eye contact, gets noticeable angry or quickly agrees, and anchored in respect and acceptance of the patient’s degree of readiness for further explorations in that moment, can help to avoid disruptions in the alliance building.

The engagement of the patient’s own agency and ability to explore, identify, process and reflect is crucial for building alliance and moving the therapeutic process towards change. Kernberg and colleagues [59] noted that interventions including interpretations are best offered when “… the patient displays some spontaneous curiosity about the nature of the interaction with the therapist, and has achieved some distance from its immediacy” (pp 104 – 105). This is indeed applicable especially to the early alliance building phase. However, even when the alliance is established and a collaborative therapeutic process with trust, respect and understanding has been formed, emerging conflicts or life events can suddenly re-evoke the patient’s resistance with reactivity, avoidance, or dismissiveness. This is especially important to keep in mind when NPD patients are struggling with reactivated internal trauma, and the subjective meaning and experience of an earlier event that have affected implicit self esteem. The patient’s withdrawal and more or less explicit rejection of therapeutic interventions can in such case indicate the reemergence of an early trauma.

### Causes of emotion dysregulation

Various underpinnings and patterns of emotion dysregulation will affect self-esteem and unfold in different ways in relationship to the therapist. Identifying the source and meaning of such primary emotional triggers is very important, sometimes crucial, for preserving the alliance and building trust and further collaboration. First*, intense emotion*, especially sudden intense aggression, self-criticism and shame can impinge on self-esteem and lead to protective self-enhancement, with enacted entitlement, or competitive or admiration seeking behavior. It can also lead to critical, aggressive or dismissive attitudes and interactive behavior. In some patients intense emotional reactions can, as mentioned above, also result in pseudo-compliance with attentiveness and protective avoidance to preserve self-esteem. Second, *compromised capacity to feel, identify, tolerate or process feelings* can contribute to intense self-preoccupation to maintain control. When such initial effort fails, the inability to integrate emotions in interpersonal contexts can result in intellectualization, intense outbursts, avoidance or withdrawal. Third, emotional experiences in the presence can relate to and activate *past psychological emotional trauma,* unfold extreme vulnerability and contribute to overwhelming shame and fear, affect intolerance or compromised ability to process the range of activated intense emotions. Such reactivations can lead to psychophysiological reactivity and withdrawal. Fourth, the *fear of exposure, failure and losing control* due to feeling emotionally overwhelmed can initiate drastic actions to preserve control, such as substance use and relapse, or suicide.

### Changes in narcissistic personality disorder functioning

Being able to actively engage the narcissistic patient in collaborative explorations can promote further awareness, but may or may not automatically lead to noticeable changes in the alliance. Real changes often occur or become noticeable in the patients’ experiences and functioning in their life outside treatment [18]. One major question when building a therapeutic alliance is whether and how the patients can gain realizations about themselves that can initiate and be applied to changes in their experiences and interactions in their outside lives [60]. Sometimes the alliance in and by itself can contribute to corrective emotional experiences that can transfer to or instigate changes in the patients’ life. Other times sudden unexpected life situations, such as progressive challenges, changes, or manageable disappointments in work or relationships, can provide noticeable motivation for and evidence of personality changes in awareness, self-regulation and interpersonal relations. [18].

However, while some patients with pathological narcissism or NPD readily move from awareness or realizations gained in the therapeutic alliance to change in outside life, others can be hesitant and dread or even fear the actual implementation of changes and the accompanying internal as well as external challenges [61]. Some may not tolerate the loss of ingrained patterns of self-enhancement, manipulation or control; others feel a strong need to avoid the external exposure that may be required, or that will more or less automatically follow a change, especially when change involve adjusting extraordinary or unattainable aspirations to personal balance, sense of genuine identity and connection, and to finding joy in life. Still others may lack motivation or interest in embracing the gain or value in shifting self functioning or patterns, especially in interpersonal interactions.

Change in patients with NPD are also related to their evaluation of own potentials as well as to the degree that their expectations and goals for treatment are reality anchored and possible to achieve. For some patients, taking ownership of competence and achievements can represent significant indications of stabilized self-esteem and decreased self-criticism, insecurity and self-devaluation. For others, realizing that high aspirations and perfectionist standards are unattainable and contributing to self-judgment and fear of failure, can be a relief, but may also cause temporary confusion, identity diffusion, and a sense of being lost in life, while searching for new and more attainable and manageable standards and goals.

A shift from self-preoccupation in the service of control, enhancement or avoidance or dismissal, toward interest in and attention to others, can be a significant evidence of change in narcissistic personality functioning. Such change can be possible when the patients have gained understanding and a sense of control, and can recognize an ability to organize, tolerate and find meaning in their own internal experiences, as well as an ability to regulate their functioning and emotions based in their sense of self-agency [62].

### Therapeutic strategies and interventions

In addition to a general and consistently exploratory, collaborative approach, the following six therapeutic strategies and interventions for alliance building have been highlighted in this paper.

First, identifying and reaching an agreement about a problem that the patient wants to address, and that is realty anchored, accessible and relevant for patient’s narcissistic pathology. This is a very important starting point for engaging the patient’s courage and motivation to address problems. It is also important for gaining a better understanding of the patient’s internal functioning, reasoning and self-esteem regulation.

Second, initial focus on self-esteem related issues rather than emotions. This can invite patients’ proactive sense of agency and encouraging further development of the treatment alliance to become solid enough to tolerate further explorations of emotional challenges.

Third, the therapist’s non-judgmental validation of patient’s subjective emotional experiences of problems and challenges. This may include an acceptance and even an initial appreciation of the patient’s narcissistic defenses and enhancement, which can be important for establishing a common ground in the therapeutic alliance for continuing unfolding of self-enhancing as well as self-undermining patterns and fear-provoking disclosures.

Fourth, gradually encouraging the patient’s curiosity and reflective ability by asking questions like “what do you think makes you react/feel/think like this” or “how do you understand this problem/event/interaction”. This serves to activate the patient’s reflective ability and self-assessment.

Fifth, engaging the patient’s sense of proactive agency by attending to and incorporating the patient’s capabilities and incentives, as well as aspirations and goals. This is especially important when the patient is struggling with underlying trauma and severe insecurity or inferiority.

Sixth, challenge self-enhancing tendencies (defenses, maneuvers, reactivity, etc.,) when the patient is able and motivated to reflect upon them. Recurrent assessments of the patients’ readiness and ability to tolerate the challenge of facing underlying vulnerability and inferiorities is an important part of alliance building.

## Conclusions

This paper has focused on alliance building with patients with NPD, which for many of those patients and their therapists can be a significant endeavor that may take months, and in some cases even dominate a major part of a long-term treatment. In some cases the alliance building can in and by itself be a main goal of the therapy. Given the NPD patients’ internal fluctuations, susceptibility to certain life contexts and events, and the unfolding of early trauma and deeper attachment patterns, every stage in the therapeutic process may activate new challenges with risks for disruptions and requirements of re-assessing, re-balancing and re-connecting, especially in the termination process. Alliance building involves recurrent testing of the different levels of relationship between patient and therapist. Those include: the real and authentic; the mutually interactive and collaborative; the unfolding transference and countertransference; the hidden unprocessed and unintegrated idealized and negative devalued aspects of the internalized relationship; the attachment patterns as they suddenly or gradually unfold; and the unfolding of early narcissistic trauma. Alliance building is present in all types and modalities of treatment. The aim of this paper has been to integrate a broad range of the accumulated facts and knowledge about narcissism, its pathology and ways of unfolding in alliance building. Strategies and interventions have been discussed that serve to protect, maintain and advance the therapeutic process and attend to emerging risks for disruption, stalemate or courses that go away from the therapeutic aim, i.e., the patient’s change and improvement in personality functioning. Of specific importance is the co-existence of and fluctuations between self-enhancement and vulnerability, which can be both overtly and covertly present, and impacting life contexts and challenging events.

An important part of alliance building is to increase the NPD patients’ awareness of and ability to identify, formulate and take ownership of their regulatory narcissistic strategies that they are enacting and perpetuating in their outside life. Whether primarily noticed in self-regulation or enacted in interpersonal contexts, or both, these strategies may for the most part be rooted in self-esteem fluctuations causing emotional reactions. However, they can also be caused by emotion dysregulation (intense emotional hyperreactivity or difficulties to identify, tolerate, process, or verbalize feelings), attachment patterns, or by overwhelming emotions embedded in trauma, all affecting the self-esteem. Clarifying whether the patients primarily has low self-esteem because of intolerance of or inability to process emotions, or whether threats to or loss of self-esteem evoke unbearable emotions, can provide a very useful awareness, both with regards to the patients’ experiences in their interpersonal interactions in general, but especially applied to the therapeutic alliance and the continuing formulations of treatment goals and strategies.

## Abbreviation

NPD: Narcissistic personality disorder
